# A Realist Explanatory Case Study Investigating How Common Goals, Leadership, and Committed Staff Facilitate Health in All Policies Implementation in the Municipality of Kuopio, Finland

**DOI:** 10.34172/ijhpm.2022.6355

**Published:** 2022-02-27

**Authors:** Maria Guglielmin, Ketan Shankardass, Ahmed Bayoumi, Patricia O’Campo, Lauri Kokkinen, Carles Muntaner

**Affiliations:** ^1^Department of Health Sciences, Wilfrid Laurier University, Waterloo, ON, Canada.; ^2^MAP Centre for Urban Health Solutions, Li Ka Shing Knowledge Institute, Toronto, ON, Canada.; ^3^Dalla Lana School of Public Health, University of Toronto, Toronto, ON, Canada.; ^4^Faculty of Social Sciences, Tampere University, Tampere, Finland.; ^5^Bloomberg School of Nursing University of Toronto, Toronto, ON, Canada.

**Keywords:** Health Policy, Local Government, Policy Implementation, Finland, Healthy Public Policy, HiAP

## Abstract

**Background:** Health in All Policies (HiAP) encompasses collaboration across government and the consideration of health in various governmental sector’s policies and decisions. Despite increasing advocacy, interest, and uptake in HiAP globally, empirical and evaluative studies are underrepresented in this growing literature, particularly literature on HiAP implementation at the local level. Finland has been a pioneer in and champion for HiAP.

**Methods:** A realist explanatory case study design was used to test hypotheses about how HiAP is implemented in Kuopio, Finland. Semi-structured interviews with ten government employees from various sectors were conducted. Data from interviews and literature were analyzed with the aims of uncovering explanatory mechanisms in the form of context-strategy-mechanism-outcome (CSMO) configurations related to implementation strategies. Evidence was evaluated for quality based on triangulation of sources and strength of evidence. We hypothesized that having or creating a common goal between sectors and having committed staff and local leadership would facilitate implementation.

**Results:** Strong evidence supports our hypothesis that having or creating a common goal can aid in positive implementation outcomes at the local level. Common goals can be created by the strategies of having a city mandate, engaging in cross-sectoral discussions, and/or by working together. Policy and political elite leadership led to HiAP implementation success because leaders supported HiAP work, thus providing justification for using time to work intersectorally. How and why the wellbeing committee facilitated implementation included by providing opportunities for discussion and learning, which led to understanding of how non-health decisions impact community wellbeing, and by acting as a conduit for the communication of wellbeing goals to government employees.

**Conclusion:** At the municipal level, having or creating a common goal, leadership from policy and political elites, and the presence of committed staff can facilitate HiAP implementation. Inclusion of not only strategies for HiAP, but also the explanatory mechanisms, aids in elucidating how and why HiAP is successfully implemented in a local setting.

## Background

**Key Messages**

** Implications for policy makers**

- Creating common goals between sectors can aid in successful implementation of Health in All Policies (HiAP) at the municipal level. Common goals can be created by having a city mandate, engaging in cross-sectoral discussions, and/or by having different sectors work together.
- Policy and political leadership for HiAP can promote positive implementation outcomes because leaders supported HiAP work and therefore provide justification for using time to work intersectorally.
- Having an intersectoral committee for HiAP can aid in HiAP implementation by providing opportunities for discussion and learning, which can foster understanding of how non-health decisions impact community wellbeing, and by acting as a conduit for the communication of wellbeing goals to government employees.
- When interpreting research findings, careful consideration must be given to context. More similar contexts may allow for greater transferability of findings.

** Implications for the public**

Health is largely determined by environments for daily living, like social and economic environments and the built environment. These environments are influenced by policies and decisions made in non-health sectors such as the sectors of education, transportation, finance, housing, labour, infrastructure, etc. Health in All Policies (HiAP) is a government policy approach that considers the impacts of health and health equity in health and non-health government policies and decisions in order to promote population health and health equity. While this is a laudable aim, HiAP is not always easily implemented. HiAP can be implemented at the supranational, national, regional, and/or municipal level. This study investigated how HiAP is implemented in a city in Finland, and found that having common goals between sectors, and having local leadership and committed staff helped improve implementation.

The importance of social determinants of health has been recognized for centuries. Nevertheless, the approach to public health has changed considerably since the 1800s, from a focus on sanitation and hygiene strategies in the Victorian age, to an emphasis on individual lifestyles as a result of advances in risk factor epidemiology in the mid to mid-late twentieth century, to an ecological, holistic approach wherein healthy public policy is sought via multi-disciplinary collaboration^1^; a shift that followed the demise of infectious diseases to the rise of chronic conditions as being the primary causes of mortality.^2,3^ This holistic approach encompassing recognition of the broad determinants of health and the need to improve health via coordinated efforts of health and non-health sectors is reflected in foundational documents since the 1970s, including the Lalonde report (1974),^4^ the 1978 Declaration of Alma Ata, the Epp report (1986),^5^ the 1986 Ottawa Charter for Health Promotion, Health in All Policies: Prospects and Potentials 2006,^6^ the final report by the commission on social determinants of health,^7^ the Marmot Review (2010),^8^ the 2010 Adelaide Statement on Health in All Polices, and the Helsinki Statement on Health in All Policies (2013).^9^

Various interventions to achieve intersectoral action for health have been coined and utilized since the 1970s, including, for instance, joined-up government, healthy public policy, and Health in All Policies (HiAP). Healthy Public Policy, which emerged in the 1980s, is characterized by an explicit focus on health and health equity in all policy areas,^10^ whereas HiAP, which emerged in 2006 as the main health theme of the Finnish European Union Presidency,^6^ provides a concrete approach to fulfilling the aim of Healthy Public Policy by systematically taking health into account. It is distinguishable from other intersectoral initiatives in that HiAP is coordinated primarily by formal structures and processes of government, and it is explicitly linked to structural or long-term governmental policies or agendas rather than being ad hoc.^11^ HiAP is “an approach to public policies across sectors that systematically takes into account the health implications of decisions, seeks synergies, and avoids harmful impacts in order to improve population health and health equity.”^9^ The HiAP approach can be implemented at various government levels, including local, regional, national, and supranational. Local implementation includes HiAP occurring within municipalities wherein governments are advantageously positioned due to their proximity to residents and the direct impact of policies and decisions.

###  Policy Context in Finland

Finland has been, and continues to be, an exemplary country in implementing and championing the HiAP approach.^12^ The idea of intersectoral action for health has existed in Finland for some time. In 1972 the Economic Council stated that many measures of preventative health policy are the responsibility of non-health public sectors.^13^ During that time period Finland also launched the North Karelia project, an intersectoral initiative, in response to high cardiovascular mortality rates. Since the North Karelia project in the 1970s, Finland has continued to recognize the importance of addressing health outside the health sector, and promote population health via intersectoral action, as demonstrated by the decision to make HiAP the main public health theme of Finland’s European Union presidency,^6^ and in Finland’s various policy documents.

The key elements in Finnish policy context that have supported systematic uptake of HiAP include the public health law and the Advisory Board for Public Health. The public health law offers a framework for intersectoral collaboration (ISC) between the health sector and other governmental departments, as well as non-governmental organizations and the private sector.^6^ The Advisory Board for Public Health coordinates the implementation and monitoring of HiAP and comprises different sub-committees for horizontal collaboration (10 of the 13 ministries represented) and for local action (various municipal and regional authorities represented).

Although Finland has had challenges in implementing HiAP, and although HiAP is a complex and deeply political process,^14^ Finland has sustainably engaged in HiAP implementation efforts^15,16^ and is arguably an exemplary country in their engagement and success of HiAP.

Despite Finland’s leadership in HiAP, and despite the growing and rich literature on HiAP in Finland^17-21^ including literature on HiAP implementation at the national level,^15,16,22^ there is a dearth of literature focused HiAP implementation at the municipal or local level. Indeed, even on an international scale the literature on HiAP implementation locally is limited, as reported in a recent scoping review.^23^

In Finland municipalities are quite autonomous and some 300 municipalities are responsible for social services and healthcare, basic education, upper secondary education, town planning, the technical infrastructure, environmental protection, and culture and sports.^18^ Of all public services, the municipalities currently cover two thirds and the state, and its regional authorities are responsible for one third. The municipalities have the authority to levy taxes, but they also receive an annual fixed amount of money from the state to fulfil their statutory duties and are involved in the drafting of national legislation.^18^ However, a major reform has currently been implemented and from the beginning of 2023 the responsibility for the organization of health and social services will be transferred from municipalities to 22 counties.

###  HiAP at the Local/Municipal Level

Municipalities within many countries globally have adopted a HiAP or intersectoral action for health approach, including Australia, New Zealand, Canada, USA, Netherlands, Denmark, Finland, Sweden, Switzerland, UK, Thailand, Israel, Brazil, Cuba, Ecuador, Iran, and Sri Lanka, among others.^23-25^ Inasmuch as there is wide variation in population size, structure, power, political representation and wealth within local governments, a wide array of HiAP mechanisms or tools are used.^25^ Governance tools for HiAP can include interdepartmental committees, impact assessments (ie, Health Impact Assessment), joined-up evaluation, financial mechanisms for partnerships, and mandates.^26,27^ Municipal HiAP governance and policy implementation may be particularly impactful on population health when compared with regional or national HiAP since many of the policies and decisions operating at this level have a direct impact on social, physical, economic, and cultural environments, and thus on the health of community residents. Several factors have been noted in the literature to facilitate HiAP at the local level, including funding, political support and local leadership, a shared vision across sectors, national leadership, a HiAP mandate, and the use of tools such as Health Impact Assessment.^23-25^

HiAP is a policy approach for improving population health^28^ and has gained traction globally. A scoping review in 2011 found 16 cases of country-level HiAP.^29^ Increasingly, municipalities are implementing a HiAP approach to improve community health and wellbeing and to address health inequities.^20,30^ As municipalities continue and begin to implement HiAP, there is a need to identify causal pathways in HiAP implementation, and understand how and why strategies for HiAP work. Shankardass and colleagues^16^ note there has been little work done to understand why certain implementation practices work in some settings. Although there has been some application of theory to HiAP,^16,28,31-36^ to our knowledge there is no published literature focusing on how and why strategies for HiAP work at the local level, and there has been no development and application of theory of HiAP implementation locally.

Therefore, given the limited research on implementation of municipal HiAP, and given the importance of municipal governance in community health outcomes, this study sought to address this gap by understanding how and why strategies for HiAP work in the municipality of Kuopio, Finland by testing hypotheses on strategies for successful implementation. Using an explanatory case study design, we sought to answer the following research questions: How is HiAP currently implemented at the local level in Kuopio, Finland? What are the underlying mechanisms facilitating successful implementation? We defined successful implementation as positive policy implementation outcomes including acceptability and feasibility of implementation across parties involved, and sustainability of the HiAP implementation process (eg, completion of a HiAP intervention activity).^36^ Assessment of positive impacts on health equity could also be considered an indicator of successful implementation; however this was outside the scope of this study. To answer the research questions, we formulated hypotheses about strategies involved in successful implementation of HiAP locally based on findings from a literature review^23^ and tested them with data on Kuopio. We used methods successfully employed previously to study complex policy implementation.^36^

## Methods

###  Study Design and Philosophy of Science

We used an explanatory case study design with a philosophy of knowledge that is realist. Case studies are most appropriate for “how” and “why” questions and investigate causal explanations,^33^ which can be done by testing hypotheses. We drew from realist evaluation methods and therefore included explication of an initial causal theory of how and why events occur. In addition to realist evaluation methods, our initial theory (in the form of hypotheses) was based on evidence from the literature on HiAP implementation locally. We focused on providing initial program theory, as per Pawson and Tilley,^37^ rather than a specific and refined theorem.

Mechanisms are at the heart of casual investigation. Such explanations can be articulated in the form of *context-mechanism-outcome* pattern configurations.^38^*Mechanisms*, which are underlying explanatory processes of a strategy/intervention (as part of a program, for instance), occur within specific social, economic, political, and physical environments.^37^ Programs like HiAP are always introduced into pre-existing social and cultural *contexts*, including particular geographical locations, and social norms and values, which are of crucial importance in explaining *outcomes,* the success or failure of a social program for instance.^37^ In our case, we were interested in testing hypotheses focused on the role of success factors for HiAP implemented identified in the literature (as described below); so we also identified aspects of the context that resembled implementation *strategies*, which we defined as intentional plans or activities related to HiAP that are involved in activating mechanisms responsible for producing implementation outcomes. Therefore, in this analysis we articulated *context-strategy-mechanism-outcome* pattern configurations, or CSMOs.

###  Hypotheses

This study was guided by findings from a scoping review, which identified several factors involved in successful HiAP implementation at the local level, including establishment of a shared vision of HiAP across sectors, and value of specific human resources for implementation including local leadership and committed staff.^23^ We devised our initial theory about how and why establishing a shared vision across sectors and the presence of local leadership and committed staff are involved in successful HiAP implementation based on evidence from the literature.^23^ To better articulate our theory, we created a set of hypotheses for each domain (Domain 1. Establishment of a Shared Vision, and Domain 2. Local Leadership and Committed Staff). (Hypotheses are presented below).

Domain 1, Hypothesis 1: Common goals between the health and non-health sector are created *(mechanism) *via win-win strategies by a government employee from the health sector to engage the non-health sector in a HiAP activity *(strategy)*, which leads to greater buy-in and/or ongoing competition of HiAP activities *(outcome)*.

Domain 1, Hypothesis 2: Common goals between the health and non-health sector are created (*mechanism)* when a government employee from the health sector learns of the other sector’s goals and objectives and when communicating with them, references those goals and objectives and uses language and terms idiosyncratic to that specific sector *(strategy)*, which leads to greater buy-in and/or ongoing competition of HiAP activities *(outcome)*.

Domain 2, Hypothesis 1: The municipality has local leadership and/or committed staff for HiAP activities who provide guidance, support, training, and/or resources *(strategy)*. Having someone help with the process makes engagement easier and less frustrating (since HiAP activities, like doing a Health Impact Assessment, can be time-consuming and cumbersome) *(mechanism)*, which leads to greater human resources and capacity to promote successful and ongoing completion of HiAP activities *(outcome)*.

Domain 2, Hypothesis 2: The municipality has local leadership and/or committed staff for HiAP activities who provide guidance, support, training, and/or resources *(strategy)*. Leaders and champions of HiAP will motivate and encourage others, and be an example to follow *(mechanism)*, which will lead to greater human resources and capacity to promote successful and ongoing completion of HiAP activities *(outcome)*.

###  Case Selection and Data Collection

Finland has a long history of using a HiAP approach, having implemented one of the earliest examples of ISC for health via the North Karelia project,^39^ and was a leader in bringing HiAP to the European and international scale when Finland made HiAP the theme during the EU presidency, which can be considered a success in consolidating rhetoric of HiAP.^40^ Kuopio has a population of about 120 000 and is a leader for wellbeing promotion, happiness, and ISC within Finland as it is one of the happiest largest cities within Finland,^41^ with Finland being reported as the happiest country in the world in recent years.^42^ Kuopio has a vision of being the “Capital of Good Life.”^43^ As such, Kuopio was selected as an exemplary case. Kuopio is also considered a mature case of HiAP – falling in the “integrated” or “institutional” stage of HiAP maturity as evidenced by a broad shared vision and political and administrative anchoring for HiAP.^32^ Semi-structured interviews were completed with ten government employees in the municipality of Kuopio between November 2018 and January 2019. Between ten and fifteen participants were sought per case in previous HiAP research using similar methods.^36^ Participants worked in various sectors/areas, including: Health and Social Services sector (1), municipal government as politicians (2), Wellbeing sector (3), Learning Services sector (1), Urban Environments sector (1), Facility Management (1), and the Traffic department (1). Key informants were identified based on a review of the literature, including grey literature, but mostly through snowball sampling. A diverse sample was sought to include participants from various sectors. Eligibility for participation was based on self-rated familiarity with HiAP implementation and Health Impact Assessment based on a Likert scale ranging from 1-very unfamiliar to 5-very familiar, with those rating themselves 3-familiar or higher deemed eligible. Of the 21 potential participants contacted, three declined due to lack of time or eligibility, and eight did not respond despite follow-up attempts.

 All interviews were in English and completed in-person in the participant’s work setting, with the exception of one, which was completed over Skype. We used an interview guide that was based on the hypotheses and included open-ended questions. The interviewer, however, included many unscripted probing questions, based on participant responses, aimed at uncovering underlying mechanisms. Questions were presented in an open-ended manner, primarily beginning with “how” or “why” an event the participant noted occurred in order to prevent leading. Moreover, in addition to asking about specific hypotheses, participants were initially asked questions like “What helps the most in promoting good working relations with employees from non-health sectors?,” followed by “how does that help?” in order to identify facilitating factors without any prompting. Participants’ responses often included mention of hypotheses, which strengthened the results. Over the course of interviews many participants gave similar responses. All interviews were completed by the principal investigator (MG). Written consent was obtained and ethics approval was granted from the University of Toronto Research Ethics Board.

An extensive review of the literature on HiAP implementation at the local level in numerous countries was completed at the outset of the study to inform hypotheses development.^23^ This scoping review identified several factors that hinder or facilitate HiAP implementation locally. A second literature review on HiAP implementation in municipalities in Finland specifically was also completed, the results of which were used as a source of data in addition to interview transcripts. The amount of literature, however, was limited.

###  Coding and Analysis 

The coding and analysis of data closely followed the methodology for realist explanatory case studies articulated by Shankardass and colleagues.^36^ Transcripts were created verbatim from the recorded interviews and included utterances and breaks. First, interviews and literature were coded for specific CSMO pattern configurations based on hypotheses and were classified as supporting, refining, or refuting hypotheses. CSMOs were compared to each hypothesis to identify if one or more were relevant, and if so, if the CSMO supported or refuted the hypothesis. New CSMOs that fell outside of the hypotheses but were relevant for the outcomes under study were also found and coded in the interviews. Transcripts were reviewed by at least two authors, and inter-rater reliability was assessed by having authors independently code transcripts and subsequently compare findings. Trial coding, as a measure taken in attempt to strengthen inter-rater reliability, demonstrated similar identification of CSMOs. Once the initial coding was completed, CSMO data were extracted and put into tables for enhanced readability. At this stage, consensus building occurred via meetings wherein at least two authors were present to review each CSMO to ensure agreement.

Subsequently, CSMO pattern configurations were then grouped by domains and patterns of similar mechanisms.^36^ Tables were created for each domain so that triangulation could be assessed to determine the level of support from multiple sources. Evidence was evaluated for quality based on triangulation of sources and strength of evidence^44^ (Table).

**Table:** Triangulation of evidence^44^

**Strong**	Thick^a^ evidence from three or more sources of data (ie, documents or different informants)
**Adequate **	Thick evidence from two source of data
**Limited**	Thick evidence from one source of data
**Thin**	Only thin^b^ evidence available
**No evidence **	No evidence was generated

^a^Thick interview/literature CSMOs entail descriptions of the mechanisms that are detailed and typically include ample description of context, mechanism, and a clear link to the outcome.^44^ ^b^Thin interview/literature CSMOs imply there is lack of critical detail about the mechanism, or there is an unclear link between the mechanism and outcome.^44^

## Results

 Results are shown by domain. As most participants described a shared vision using the term “common goal” or “common target,” the domain was renamed to reflect this evidence.

###  Common Goals 

 Our hypothesis about the importance of having or creating common goals in the implementation of HiAP was supported by strong evidence (thick evidence from six sources of data). However, findings suggested modifications to our initial hypotheses. For instance, we found limited evidence of the use of win-win strategies and using common language as necessary strategies for creating the mechanism of common goals and promoting implementation. Instead of these strategies, data revealed the use of three other strategies for enabling the mechanism of creating common goals that were not included in our initial hypotheses, including the Kuopio Strategy until 2030 (hereafter referred to as the Kuopio Strategy), communication across sectors, and collaboration together across sectors.

The municipality of Kuopio has a city mandate document, the Kuopio Strategy,^43^ which is used by organizations and government sectors to guide their decisions/policies/projects; it includes strategic priorities in the areas of business, education, environment, wellbeing, etc. Participants discussed the Kuopio strategy as a facilitating factor for HiAP implementation since it aided in providing various sectors with common goals. A politician identified the Kuopio Strategy as being helpful for getting various sectors to work together (outcome), and when asked why it was helpful he responded by saying, “because if we think leading of city, city is not just organization, it’s a community, so if we have this kind of*…*mind map*…*if they have that all our work companies and peoples thinks that we have to do this kind of things, and that we goal*…*same goal.” It was also noted that while “it’s quite a difficult process and it takes a few years” to get people from various sectors to work towards common goals, there is an already established culture of ISC, which likely aided in creating a favourable environment for HiAP (context).

There was much agreement on the importance of having discussions with personnel from other sectors as a prerequisite for understanding and subsequently having/creating a common goal. Via discussions, sectors gained understanding of the other sector’s goals and what they do, which enabled the creation of common goals. When asked what helps the most in promoting good working relationships, a participant from the Health sector identified having a common target. When asked “how do you have common targets?,” she responded, *“*we create them together*…*we cannot tell them that do something because we want them to do. It’s not very good, so that’s why we have to discuss and find the common targets.” This allowed her to work successfully with non-health sectors and led to the consideration of geriatric needs in planning and services in non-health sectors (outcome).

Additionally, in some instances, participants noted that discussions occurred when working on a project with another sector (collaboration together across sectors). Through discussion with other sectors (specifically health with non-health sectors), non-health sectors gained understanding of how their sector-specific decisions, policies, and actions impact the health of Kuopio residents. It was through these understandings that sectors were able to develop common goals, which ultimately led to enhanced intersectoral work to promote health and wellbeing. A participant from the Urban Environment sector described his experience working with the health sector on a project together as “speaking with the professionals like health sector people and they have like teach and we have had common…that I have figured out that we have same goals, that we can do together these.” An important consideration of collaboration across sectors and discussions as facilitators for successful HiAP implementation is that meetings and discussions seemed to mostly occur in-person and not online. In an examination of HiAP in a community in Denmark, Christensen and colleagues^45^ found that face-to-face interactions promoted collaboration, and fostered engagement and involvement. While only one participant commented on the benefits of being in-person (saying it is easier to discuss and explain your goals and objectives), and although it is beyond the scope of this research, it is worth contemplating if the meetings discussed by various participants would have been so successful if they had not been in-person.

###  Local Leadership and Committed Staff 

In Kuopio, Finland, local leadership and committed staff appear to be important strategies in successful HiAP implementation via policy and political elite leadership and the wellbeing committee, as supported by strong evidence from four sources of data. Local leadership can include impetus for HiAP efforts by local actors including policy and political elites. These elites are actors who have been granted some form of formal authority by which they can exert control over the policy and political processes and its outcomes.^16^ Committed staff included personnel who were enabled to engage in and committed to intersectoral work alongside their other duties.Notwithstanding the supporting evidence, many aspects of our initial hypotheses veered from study results. For instance, almost all of the participants had not received formal training for HiAP; rather, learning to work intersectorally to promote health was learned on the job and happened because it was part of the working culture. Moreover, we did not find any evidence confirming that local leadership and committed staff make engagement easier; instead policy and political elites provided support and justification for HiAP work (mechanisms). Lastly, our hypotheses did not anticipate the importance of the intersectoral committees/groups, like the wellbeing committee.

 Support for HiAP by policy and political elites existed in various forms. One participant noted that having the vice mayor support HiAP affected implementation because it allowed her to have common goals with leaders, and the vice mayor supported her sector’s goals. She said without the vice mayoral support “it wouldn’t happen.” She went on to describe how the city could not have had the target of being an age-friendly city (outcome) without political support. Another participant noted that many bosses (eg, departmental/sector managers) and leaders in Kuopio believe that cooperation is a good thing and tell their employees they must work together. When asked why that helps in promoting ISC, she responded by saying because she can use her work time to work intersectorally. It seems that support from policy and political elite leaders provides justification for employees to engage in ISC. Counter-factual evidence for this is provided via another participant who, unlike the majority of participants, described her sector as working in a silo (this participant was in a different sector from the six service sectors where the majority of participants worked). She explained how ten years ago her department had a director who was interested in ISC and active in the World Health Organization (WHO), but since he has gone things have gone “backwards” in intersectoral cooperation and HiAP thinking.

 Evidence was found in support of the strategy of intersectoral committees/groups, including the wellbeing committee, in the successful implementation of HiAP. Having a wellbeing committee seemed to enable discussions between sectors and provide opportunities for non-health sectors to learn about the ways in which their decisions and actions affect health (mechanism). A participant described how the wellbeing committee is made for “all activities which are outside the box of health and social care…activities which are promoting health and wellbeing.” Another participant discussed working with the environment unit in an intersectoral group during which she was able to convince the unit that wellbeing promotion was part of their job, specifically, in building schools. She brought up the issue of having short doors in washrooms, which enables students to take pictures of each other and post them online, a form of sexual harassment. She helped them realize wellbeing promotion was part of their job (mechanism), which led them to incorporate student wellbeing in the design of the school (outcome). The wellbeing committee was also helpful in HiAP implementation because each sector leader is informed of the wellbeing goals, which are then conveyed to other government employees. One informant noted how her boss “brings so much information to us…she always says now these are the goals for next year and these are the statistics that I got from research…it’s like mutual understanding and sharing ideas and so if she says there’s this certain problem in the city now…we discuss together that maybe we should do it that way or I have an idea.” This made the goals “easier” and more “understandable.” This quote demonstrates not only how leaders facilitate HiAP implementation via communication of information, but also illustrates useful departmental leadership skills. The sector leader presented the goals with research and rationale, and used an open approach whereby solutions were created together with employees in her sector.

## Discussion

 Our case study of Kuopio, Finland, based on interviews from a diverse group of government employees, provides strong supporting evidence for the hypotheses that having common goals between sectors, and that local leadership and committed staff, facilitate intersectoral work for health. Through obtaining and testing data related to our a priori hypotheses, we found support for aspects of our original propositions, but also learned of nuances that differed from our predictions.

We found ample evidence supporting the presence or creation of common goals between sectors for successful HiAP implementation locally. This finding aligns with results of a scoping review on HiAP in municipalities that found lack of a clear vision or objective was a barrier for HiAP.^24^ Similarly, Larsen and colleagues,^46^ based on their analysis of intersectoral action of health in a Danish municipality, conclude that common goals are needed to incentivize collaboration. A common goal can be created by having a city mandate that promotes the tenets of HiAP (ie, wellbeing promotion, awareness of social determinants of health, etc), or by having different sectors work together on projects or planning. A clear mandate such as an official policy legitimizes ISC for health and can facilitate intersectoral action.^25^ Having discussions with individuals from another sector, which leads to greater understanding of that sector and enhances relationship building, can also result in common goals. It was discovered that via discussions with the health sector, some non-health sector employees came to realize the importance of their sector in influencing health and wellbeing. After this realization occurred, non-health sector employees incorporated health and wellbeing in their sector-specific planning and actions. Baum and colleagues^28^ similarly found that collaboration on HiAP in South Australia was helpful in increasing understanding of social determinants of health in public servants from a wide range of departments.

Policy and political elites and the wellbeing being committee, examples of local leadership and committed staff, were also revealed as facilitating factors in HiAP implementation. Participants noted HiAP would not happen without political support, and having this senior level support was required to give employees permission to use their time to engage in ISC. Greer and Lillvis^47^ likewise found that HiAP objectives given by political leaders justified actions that otherwise might have been neglected. Political will has been noted as an important factor in intersectoral action for health locally,^25,48^ and lack of political will has been documented as a hindering factor.^43^ The wellbeing committee enabled discussions, which led to learning and understanding (specifically that non-health sectors understand how their decisions impact health), and acted as conduit for departmental bosses to learn about the wellbeing goals and inform intradepartmental colleagues.

 While results support the basic tenets of our a priori hypotheses, many aspects were different from original predictions. For instance, despite strong evidence in support of having/creating a common goal for the first domain, the strategies for creating common goals varied. Whereas we predicted using terms idiosyncratic to other sectors to be an important strategy, we found only some evidence for this. We had not hypothesized that the Kuopio Strategy would be an important determinant in creating common goals. With the second domain, although we found support for the strategy of local leadership and committed staff, we found a broader set of related mechanisms than we proposed. For example, we had anticipated that leaders would facilitate HiAP implementation by motivating and encouraging others; instead, we uncovered that leaders were important in implementation by providing justification for HiAP work and because their support for the aims of HiAP was viewed as a requirement for successful implementation. Moreover, while both domains were supported with evidence, more data supported the first domain, and many participants noted the importance of a common goal between sectors without any prompting during the interviews.

Few studies have investigated strategies and the underlying mechanisms (how and why the strategies worked) for HiAP implementation locally. To our knowledge, there has been no literature published focusing on how and why HiAP is successfully implemented in a Finnish municipality, even though Finland has been a pioneer in and champion for HiAP. Moreover, we are unaware of any literature focused on uncovering mechanisms for HiAP implementation strategies operating on the local level. Uncovering causal mechanisms and not simply describing associations provides insights on effective solutions.^49-52^ For instance, an association between the *strategy* of an intersectoral committee (ie, the wellbeing committee in Kuopio) and the *outcome* of successful HiAP activities may not provide the necessary information needed to successfully implement HiAP elsewhere. Since the *mechanism* by which this strategy is effective includes discussions between personnel from various sectors that enable increased understanding of how non-health decisions and policies impact health, an online committee using a top-down approach whereby members are not given an opportunity to converse with each other could result in disappointing outcomes. As many employees are currently working from home amidst the coronavirus disease 2019 (COVID-19) pandemic and intersectoral meeting structures may be varied, such consideration may be particularly helpful. Lastly, intersectoral committees have been noted to be ineffective in some cities in Denmark.^48^ Holt and colleagues^48^ noted that, among other factors, public health teams were infrequently able to translate their public health aims into relevant boundary issues that would encourage strong buy-in. It would be a worthy future endeavor to investigate the contexts and mechanisms of both effective and ineffective intersectoral committees.

Finland’s dominant political patterns and policies over the last decades are important contextual factors in the country’s implementation of HiAP. Finland has a long history of egalitarianism and falls within the social democratic welfare regime type. A principle socio-political policy in Finland is the welfare state, which includes a system of progressive taxation, comprehensive social security to protect citizens in the event of illness or unemployment, support for families with children, and free education and vocational training.^18^ Although there are increasing pressures of globalization and neoliberalism, there is relatively less inequality in Finland compared with other liberal countries such as Canada, the United States, and the United Kingdom.

There have been instances of successful programs in one area of the world being applied elsewhere with disappointing outcomes; often the contextual factors were not adequately considered.^53^ While generalizability of one case can be limiting, in-depth analysis of mechanisms and contexts can permit such research to be used in theory building. Moreover, while careful consideration of findings and relevant contextual factors should be employed, our findings can be used strategically by decision makers in HiAP implementation in their localities. Specifically, municipalities in high-income countries of liberal, democratic institutions in the process of adopting or implementing HiAP may consider utilizing similar strategies while aiming to create auspicious environments or contexts for success. Moreover, municipalities with already established good working relationships between people from different sectors, and those with smaller population sizes may particularly find Kuopio’s approach to HiAP applicable.

 A notable strength in our study is the use of multiple researchers in coding, analysis, and triangulation. Transcript coding was confirmed by two authors on two separate occasions (at two different steps in the analysis), and triangulation was used to assess the strength of evidence from multiple interviewees and literature. Additionally, we sought to uncover explanations of how and why strategies for HiAP work, which aids in untangling of the intricacies of the policy implementation process.

There are some limitations to our study. While we included a diverse group of informants, we did not interview anyone from the business/economic sector, which may have provided us with different insights. Such insights may have been particularly interesting since the finance sector has been noted to have ideological conflicts with HiAP tenets and be absent in participation in HiAP projects.^15,54^ This sector may be particularly resistant to engagement in HiAP, despite their important role in influencing social determinants of health. While a translator was available to translate some Finnish documents to English, the primary investigator cannot read Finnish and therefore some Finnish documents may have been missed. Interviews were done in English, which could have excluded actors who did not feel comfortable expressing themselves in English. However, we speculate that the views of those more fluent and comfortable in English are not likely to have been different. Additionally, our approach did not allow us to explicitly assess the importance of different outcomes objectively (eg, if the gains were small vs. large). Finally, the short time allotted for interviews (one hour) limited our ability to explore other context-mechanism-outcomes.

## Conclusion

 Understanding how and why strategies for HiAP at the local level work, and the relevant contexts in which they occur, may be of immense benefit for those wishing to successfully apply HiAP in their region. Moreover, the accumulation of such knowledge creates a foundational base in this growing field, which can be used to build and test theory on HiAP implementation locally. While literature has been published on HiAP implementation at the national level, there has been little focus on municipalities, the level where many social determinants of health have a direct impact on the health of local residents. Our study provides useful insights into the strategies and mechanisms facilitating HiAP implementation in an exemplary municipality. We conclude that having a common goal, local leadership, and committed staff can be important factors in successful intersectoral action for health in municipalities in the context of a mature HiAP setting.

## Ethical issues

 Ethics approval was granted from the University of Toronto Research Ethics Board.

## Competing interests

 Authors declare that they have no competing interests.

## Authors’ contributions

 Conception and design were completed by MG, CM, KS, and AB. Data was acquired by MG. All authors participated in the analysis and interpretation of data, drafting of the manuscript, and critical revisions. Funding was obtained by MG and CM. Administrative, technical, and material support was provided by MG. Supervision was provided by CM, KS, and AB.

## Funding

 This work was supported by funds from the University of Toronto.
